# Explaining workers’ inactivity in social colonies from first principles

**DOI:** 10.1098/rsif.2022.0808

**Published:** 2023-01-04

**Authors:** Moein Khajehnejad, Julian García, Bernd Meyer

**Affiliations:** Department of Data Science and Artificial Intelligence, Faculty of Information Technology, Monash University, Clayton, Victoria, Australia

**Keywords:** social insects, social colony, lazy workers, task allocation, evolutionary game theory

## Abstract

Social insects are among the ecologically most successful collectively living organisms, with efficient division of labour a key feature of this success. Surprisingly, these efficient colonies often have a large proportion of inactive workers in their workforce, sometimes referred to as *lazy workers*. The dominant hypotheses explaining this are based on specific life-history traits, specific behavioural features or uncertain environments where inactive workers can provide a ‘reserve’ workforce that can spring into action quickly. While there is a number of experimental studies that show and investigate the presence of inactive workers, mathematical and computational models exploring specific hypotheses are not common. Here, using a simple mathematical model, we show that a parsimonious hypothesis can explain this puzzling social phenomenon. Our model incorporates social interactions and environmental influences into a game-theoretical framework and captures how individuals react to environment by allocating their activity according to environmental conditions. This model shows that inactivity can emerge under specific environmental conditions as a by-product of the task allocation process. Our model confirms the empirical observation that in the case of worker loss, prior homeostatic balance is re-established by replacing some of the lost force with previously inactive workers. Most importantly, our model shows that inactivity in social colonies can be explained without the need to assume an adaptive function for this phenomenon.

## 1. Introduction

Social insects live in large complex societies and enjoy remarkable ecological success [1,2]. This is often attributed to their ability to allocate their workforce adaptively in response to changing environmental demands [3]. It may thus come as a surprise that social insect colonies commonly harbour large sub-populations of inactive workers which can comprise up to half their populations [4]. The existence of these inactive workers is counterintuitive in populations that have evolved to very high efficiency [5]. It is unclear what is the reason behind this. Shedding light on this phenomenon may have far-reaching implications for understanding insect societies in general, and their mechanisms of task allocation in particular.

Research on inactive workers has focused primarily on empirical work to establish the existence of the phenomenon. There is little theoretical or modelling work to establish the causes or general principles behind it. Here, we propose that inactivity in large colonies may arise as a by-product of learning and interactions with the environment depending on the ecological conditions. This alone can explain variation in levels of inactivity across different ecological settings.

We consider a task allocation scenario [6–8]. Every colony has to balance a complex set of tasks, spanning foraging, brood care, defence, nest maintenance, thermoregulation and others. Task allocation and task choice in social insect colonies are key features of the organization of insect colonies and pivotal to ensure their survival and ecological success [9–11]. It is well established that the process of task allocation normally responds to altered environmental conditions such as food resources abundance, predation threats and climate conditions [12,13].

On the one hand, it has been established that morphological and genetic factors play a strong role in determining which individuals will perform which tasks [14–16]. On the other hand, it is also established that faster and self-organized mechanisms for task allocation exist that allow the colony to react quickly and flexibly to changing task demands that may be driven by changes in the environment or in the colony itself [7,17].

The fast dynamics of the latter type of workforce allocation is driven by the interplay between workforce distribution, interactions structures and environmental factors [18–22]. In particular, many empirical studies underline the role of social context and interactions in the task choice of individuals [20,23,24].

Interactions can convey knowledge about the state of the colony and provide cues for behaviour and a source for social learning [25]. Individuals have limited or no knowledge of the global state of the colony and have to make behavioural choices based on local information that is directly accessible to them [12,26]. Local information from interactions with other individuals and the environment can often serve as a proxy for more global colony states. For example, the number of workers willing to receive food from incoming foragers can serve as an indication of the overall nutritional situation of the colony [27,28]. Like the slower dynamics, driven by morphological and genetic factors, the fast self-organized task selection, reliant on interactions, may cause workers to become specialized in certain tasks or even to develop preferences towards inactivity [19,29].

We present a formal model of individual-level task choice behaviour that integrates these interactions and the influence of environmental factors to explain inactivity. Our modelling approach is based on evolutionary game theory (EGT) [30], which describes how a group of individuals adapt their behaviour based on interactions with others and the environment. To do so, we formulate a task allocation game in which benefits arising from tasks performance are shared among the colony while each worker bears the individual (metabolic) costs of their actions. The ensuing learning dynamics allows us to derive conditions under which inactive sub-populations are likely to emerge.

Our model leads to two main insights: (i) the existence of inactive workers may be an epiphenomenon arising from task allocation mechanisms that has no adaptive function in and of itself; (ii) whether inactive subgroups emerge in a population may be driven by the environmental conditions, exactly because of the adaptivity of self-organized task allocation to the environmental conditions. The exact same self-organized colony organization may prevent inactivity in one type of environment but cause it to emerge under different environmental conditions. This opens up an interesting complex of questions about the evolutionary development of self-organized task allocation.

Furthermore, we show that the emergence of inactivity may be limited to scenarios beyond a certain level of minimal task choice complexity. Potentially, this has important implications for experiments. This observation can be interpreted as guidance to not reduce the task choice complexity in experimental assays too far, because radically stripped-back assays may cause the disappearance of the very phenomenon that is to be observed.

The rest of this paper is organized as follows: §2 describes the background empirical literature as well as related modelling approaches; §3 presents the model, and its results (simulations and analytic) are discussed in §4. We conclude in §5.

## 2. Background

### 2.1. Free-riders in social organizations

Inactive sub-populations are a widespread phenomenon in collective behaviour, from active particles to insects, animals and humans [31,32]. In some cases, such inactivity may actually serve a functional role; for example, it may be due to physical constraints of the work or the environment [33]. However, this is not always the case. The ‘free-rider’ argument, originally proposed by Mancur Olson [34,35], suggests that, in any group of rational actors working for a common good, a part of the population may choose not to engage in the collective action. The main reason for this is that individual and group incentives diverge in certain settings. In the presence of shared benefits, an individual may receive a higher individual benefit if it forgoes the cost of contributing to the common good, provided it is still allowed to benefit from it. In such situations, individuals are incentivized to become free-riders [36], and *rational* individuals will not participate in the collective action unless they receive some side-payment [34]. Rationality, in this context, is taken to mean that individuals seek to maximize their expected individual payoff and that they can assess this payoff correctly. The situation may be complicated by the fact that benefits are often only obtained through kinship or as by-products [37,38].

Free-riding is also observed in human society. Immediately obvious cases include fare evasion in public transport systems [39], loafing in large learning groups [40] and others. On a larger scale, it has been shown that banking systems [41], large cooperations [42] and large industry collaborations [43] are all prone to free-riders. An increased group size is associated with the emergence of free-riding [36,44,45]. One reason that has been put forward to explain this is that large and heterogeneous social groups often do not monitor their cooperatives effectively [46]. In human societies, resilient public goods are often supported by a minority of contributors, which implies free-riders are ubiquitous even around successful cooperation [47,48].

Free-riding is also widely observed in animal societies [49,50]. A typical example are non-breeders in cooperatively breeding groups, such as carrion crows (*Corvus corone corone*) [51], paper wasps (*Polistes dominulus*) [52] and digger bees (*Centris pallida*) [53]. Similar free-riding has been observed in crickets and frogs [54]. Other examples include African lions (*Panthera leo senegalensis*) which tolerate group members that appear to safely hang back in the presence of a threat [49].

Outside of the social insects context, the general question how such free-riding may coexist in the context of collective action has been studied widely using multi-player games [44,55]. Paradigmatic models include the snowdrift game [56], its *n*-person variants [57] and one-shot games with two strategies [58]. Game-theoretical models have also been used to study mitigating influences, such as individual recognition [59,60].

The application of game-theoretic models to animal groups has focused on the evolutionary origin of behaviours [61] rather than the learning of behaviours, with which we are concerned here.

### 2.2. Empirical work on social insects

Uneven distributions of workload have been widely observed in social insects [2,52,62–64]. Social insect colonies, such as bees, termites, ants and wasps, can comprise in excess of 50% inactive workers at any one time [65]. The prevalence of these inactive individuals is particularly surprising in eusocial insects since their genetics suggests that the collective benefit plays a much stronger role than individual benefit [6].

It has been suggested that passive individuals may provide group benefits that are not immediately apparent. The main hypothesis is that inactive workers form a *reserve workforce* that can quickly be activated in times of unexpectedly high task demands or sudden worker loss, therefore, providing colony flexibility and resilience [4,62]. This could be driven by individual variation in worker response thresholds [66] and there are some empirical findings in *Temnothorax rugatulus ants* [2,65] that are consistent with this idea. Recent work on the evolution of task allocation [67] has generalized this idea and made it more precise by presenting a mathematical model at the level of ultimate causes. This lends support to the idea that partial inactivity may allow the system to adapt more rapidly in some types of dynamic environments. The benefits that would be derived from such a reserve workforce have, to the best of our knowledge, never been quantified, neither empirically nor through modelling. It is thus impossible to say if and when such benefits would outweigh the cost of biomass production and maintenance of inactive workers and overall provide a fitness benefit, and various empirical studies question this hypothesis [68].

The idea that partial inactivity in a colony may arise without an adaptive function has been advanced before. Charbonneau & Dornhaus [23] proposed that differences in activity levels, including inactivity, may be either a limitation of the evolved group-level organization or an adaptive feature. Their paper presents a comprehensive conceptual discussion of possible reasons. It is worth noting that these two explanations are not mutually exclusive but provide alternative hypotheses which may apply to different ecologies.

### 2.3. Models at the proximate level

Despite the empirical evidence, inactivity in social insect colonies has not been the explicit focus of many modelling efforts at the level of proximate mechanisms. We believe this is problematic, and that models have a larger role to play in guiding empirical research and moving beyond hypothesis. A few notable exceptions are discussed next.

One recent study demonstrates how inactivity may arise based on a threshold model [62]. While individual thresholds provide a plausible proximate mechanism, no connection to ultimate causes is drawn. Closest in spirit to our work is a recent compartmental model that studies how task allocation and passive workers arise from the interactions between individuals [68]. While this work primarily focuses on the influence of colony size, we are focusing on the influence of environmental factors. In common with this study, we are investigating whether interaction alone can drive task allocation patterns that promote inactivity. Focusing on the interaction dynamics allows us to investigate an important alternative explanation: that inactivity may not have an adaptive function and ultimate cause at all, but that instead it may simply arise as a (non-adaptive) by-product of the decentralized process by which task allocation is regulated.

The influence of environmental factors and ecologies on evolutionary trajectories is beyond doubt. Similar influences can also shape the dynamics of social group behaviour caused by learning on lifetime scales in much more complex ways than one might expect at first. Game theory promises to provide an excellent handle on these questions, as it directly captures the cost involved in producing a common good. Environmental factors, such as the difficulty of foraging, can thus be immediately included in a behavioural model, and their influence on the lifetime dynamics of group behaviour can be investigated with the established mathematical toolbox of game theory.

Here, we integrate these aspects into a game-theoretic model to advance a parsimonious hypothesis for free-riding in social insect colonies: the combination of ecological factors and task allocation dynamics are enough to let inactivity in colonies emerge.

### 2.4. Evolutionary game theory as a model of change in ecological timescales

We arrive at our conclusions based on an EGT model [69]. In this kind of model, agents follow simple rules to adjust their behaviour in response to an environmental signal, typically known as payoff [70]. EGT tracks dynamically how a population responds to environmental changes, by considering individuals and their interactions [30].

While originally conceived to model changes across evolutionary timescales, with payoff akin to fitness, this conceptual framework is agnostic about the timescale of change. EGT is also often used to model learning and faster processes that involve change in an ecological timescale, and where payoffs are interpreted as feedback signals and not fitness [71,72]. This framework is ideal to capture how agents actively adjust their task choices based on task performance experience and environmental factors when cooperating on multiple tasks.

In our model, given that change is driven by individual behaviours, we do not aim to make an evolutionary argument. Such an argument would be problematic given the discussion around the unit of selection and social insects [73,74]. Instead, our model should be interpreted as learning in ecological timescales within a social organization arrangement that is already in place via selection at a higher unit of selection. One possibility is that mechanisms of social learning have been selected for in the context of other functions and that the partial inactivity observed is just a by-product of these learning mechanisms.

Our approach is related to that of Doebeli *et al.* [56], but allows for a more complex game with nonlinear payoff functions grounded in the behavioural ecology of social insects [6]. It also allows for the crucial element of inactivity, previously ignored in other nonlinear games [75].

## 3. The model

**Figure 1.**
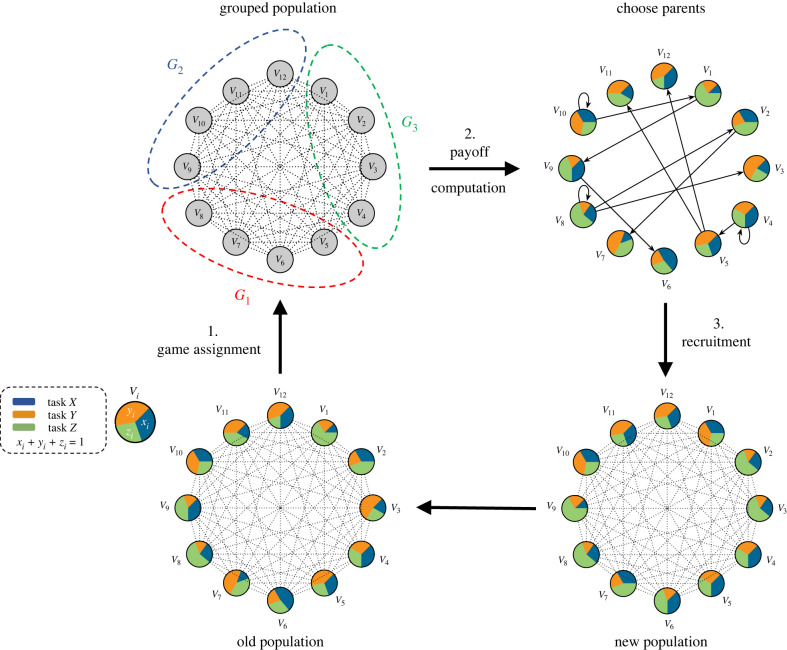
Schematic diagram of different steps in the social learning paradigm from an EGT-based perspective. Each circle represents an individual in the colony and its division into three colours indicates the proportion of engagement in each of the three tasks or its response traits *x*, *y* and *z*. The dotted lines connecting all possible pairs of individuals mimic a well-mixed population where any arbitrary pair of workers can potentially interact with each other. After the first step of the evolutionary process (1. *game assignment*), the population is divided into different games (*G*_1_, *G*_2_ and *G*_3_). The payoffs of each individual are then obtained in the corresponding games (2. *payoff computation*). The calculated payoffs form the basis for choosing successful individuals to copy. Each directed connection at this stage originates from the selected worker and ends at the individual who is copying the traits of that individual. This step is then followed by 3. *recruitment* of these new trait values. This new population with the same individuals who are now representing new trait values goes through the same process in a loop until the steady state is reached.

We briefly summarize the core of the EGT framework. We assume a finite size population *P* of agents in which each agent is completely characterized by a set of trait values. The model proceeds in discrete time steps. In each step, agents interact in groups of fixed size *n* (‘*n*-player games’ in EGT terminology) and each agent *i* receives a payoff $\Pi_{i}$ from this group interaction. Π generally depends on agent *i*’s own trait values as well as those of the other agents in the game. Agents learn from each other by imitation, for example by adopting the traits of another agent. Note that this can be interpreted as caused by recruitment as well as imitation. If the recruitment effort is modulated by task performance experience, successful agents will be more readily imitated. Updating the traits for each agent is the final step in an iteration before the next round starts with the formation of new interaction groups. Figure 1 depicts the overall schematic of the dynamic process of the system. This simple but powerful model can be interpreted as an evolutionary process (where trait copying captures genetic propagation) or as a behavioural process (where trait copying captures recruitment or other forms of entrainment and social learning).

We start our investigation from the simplest possible task allocation problem that only involves the choice between two prototypical tasks (which we will call *X* and *Y*). Each worker is characterized by the probability with which she engages in a particular task. Since we are interested in the emergence of inactivity, we additionally include the possibility to not pick either task. Formally, agent *i* is thus characterized by a triple (*x*_*i*_, *y*_*i*_, *z*_*i*_) where $x_{i}\in R$ and $y_{i}\in R$ are the probabilities to execute the first and second task, respectively, and $z_{i}\in R$ the probability to remain inactive. As $\forall i\;:\;\;x_{i}+y_{i}+z_{i}=1$, we can model a population of *N* workers as a two-dimensional vector of trait values (*x*_*j*_, *y*_*j*_)_*j*=1, …,*N*_. Since task choices are made in discrete steps, the trait values *x* and *y* can also be interpreted as the average fraction of effort invested into the first and second task, respectively, while *z* is the ‘forgone effort’ or the average degree of inactivity.

Individuals interact in groups of *n* individuals. In EGT parlance, the population constitutes *K* separate *n*-player games *G*_1_, *G*_2_, …, *G*_*K*_, where *K* = *N*/*n*.

In social insects, benefits resulting from task execution are shared and depend on the collective effort invested rather than only on individual effort. A key feature that we wish to explore is task combinations where multiple tasks need to be performed by an appropriate number of workers for the colony to function well. We model this with a multiplicative coupling of benefit *B*_*X*_ of Task *X* and *B*_*Y*_ of Task *Y*.

3.1 $$B(X_{k},Y_{k})=\frac{1}{n}\;B_{X}\left(\sum_{x\in X_{k}}x\right)\cdot B_{Y}\left(\sum_{y\in Y_{k}}y\right).$$

Here $X_{k}$, $Y_{k}$ are the collective engagement levels of all individuals in *G*_*k*_. The direct and immediate cost of executing a task, on the other hand, are borne by the agent performing the task and depend on the individual effort invested. Costs for multiple tasks are additive.

3.2 $$C(x_{j},y_{j})=C_{X}(x_{j})+C_{Y}(y_{j}).$$

The payoff for individual *j* participating in game *G*_*k*_ is given as the difference between benefit obtained and cost incurred. In game *G*_*k*_ individual *j* thus receives a payoff $\Pi_{\;j,G_{k}}$ as

3.3 $$\Pi_{\;j,G_{k}}=B(X_{k},Y_{k})-C(x_{j},y_{j}).$$

The characteristics of the tasks and the environment are captured in the shape of the cost and benefit functions.

For the purpose of our discussion we pick two task types with contrasting characteristics: (i) a task with concave benefit shape and (ii) a task with sigmoidal (thresholding) benefit shape. Regulatory homeostatic tasks, such as thermoregulation, are good examples of the first type: a certain amount of effort is required to keep the colony at the optimal temperature and the optimum benefit is obtained when this effort is invested. Further effort cannot improve the benefit and may, in fact, decrease it; for example due to overcooling by fanning or overheating by incubation [76]. Defence is a good example for the second type: below a certain effort no benefit is obtained (the conflict will ultimately be lost [77]). Similar considerations hold for brood care: below a certain level of effort the brood will not thrive. Once the level required to sustain the brood is exceeded, the effort invested determines the growth rate of the brood, which levels off at a certain point [78]. We assume marginally decreasing cost functions for both tasks. This reflects the fact that tasks commonly become easier with practice, i.e. when more effort is invested.^1^

Many other function shapes are relevant for other task types and can be analysed in a similar way. For the purpose of this initial discussion we shall focus on these two types.

As we are interested in how environmental influences impact on task allocation, we parametrize each benefit function such that the hardness of the task (i.e. the benefit obtained for a fixed amount of effort) can be scaled. *b*_2_ captures how difficult the first task is, e.g. for thermoregulation it is directly related to the ambient temperature. Two further parameters determine the difficulty of the second task: *w* (inflection point) and *β* (slope).

**Figure 2.**
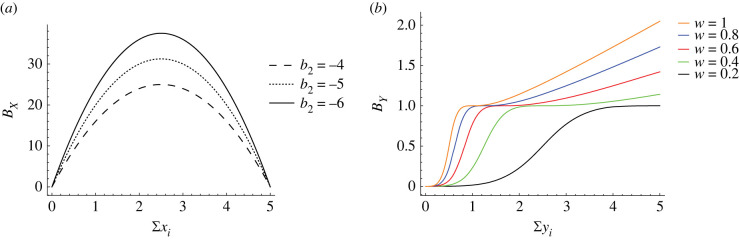
Benefit functions of tasks *X* and *Y*. (*a*) Example benefit functions for a homeostatic task *X* with different *b*_2_ components. The plots show the collective benefit as a function of group effort. (*b*) Example benefit functions for task *Y* where parameter *w* is altered at *β* = 3. The plots show the collective benefit as a function of group effort. The behaviour of the benefit function for *w* = 0.2 shows a purely thresholded pattern.

**Figure 3.**
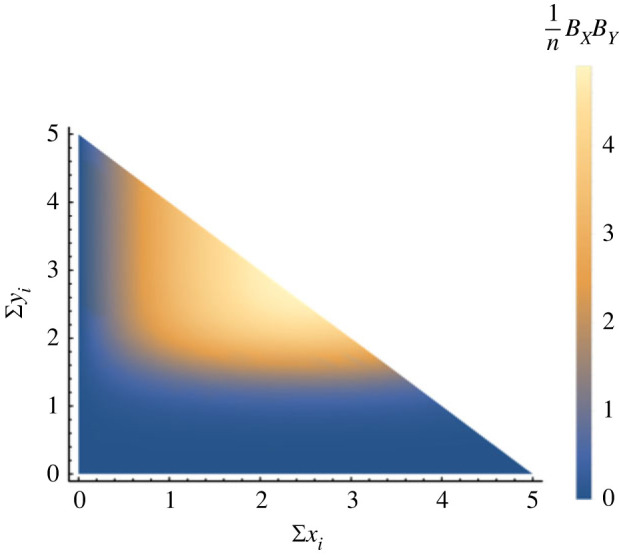
Example benefit functions for the system with all three tasks *X*, *Y* and *Z* embedded. Individual benefits are shown as a function of collective group effort. Here we have: *b*_1_ = 20, *b*_2_ = −4, *w* = 0.3 and *β* = 3, which is representative of a homeostatic *B*_*X*_ and a thresholded *B*_*Y*_ (please refer to electronic supplementary material, appendix S1 for more details on these parameters).

The shapes of example benefit functions are shown in figure 2, the combined benefit for both tasks is shown in figure 3. These functions are insured to stay non-negative for all parameter sets. Full details are given in electronic supplementary material, appendix S1.

We focus on a basic form of social learning through recruitment assuming that the recruitment signal is modulated by the task performance experience. We implement this as an individual-based, discrete time process. At each time step, the population is randomly divided into *K* *n*-player games and all individuals receive payoffs according to their trait values and the composition of the interaction groups they are participating in. Subsequently, individuals recruit others with a probability that depends on their performance relative to the population average: $e^{\alpha \Pi_{l}}/\sum_{m=1}^{N}\;e^{\alpha \Pi_{m}}$. Each trait value may also undergo small variation akin to mutation: $x_{j}^{t}\leftarrow N(x_{j}^{t},\sigma )$, which is to be interpreted as autonomous behaviour exploration by variation in the context of learning. Full details are given in algorithm 1. Its structure follows the standard game-theoretic implementation of replicator dynamics for an *n*-person game [80]. Drawing the games from a larger population should primarily be seen as the implementation of a stochastic sampling process rather than representing a direct agent-based implementation of a biophysical process. However, it could also be interpreted as such, assuming agents interact in specific tasks in smaller *n*-player groups within a generally well-mixed population. Such well-mixed populations are a standard assumption for many models of insect behaviour, in particular, the paradigmatic threshold reinforcement model [81].


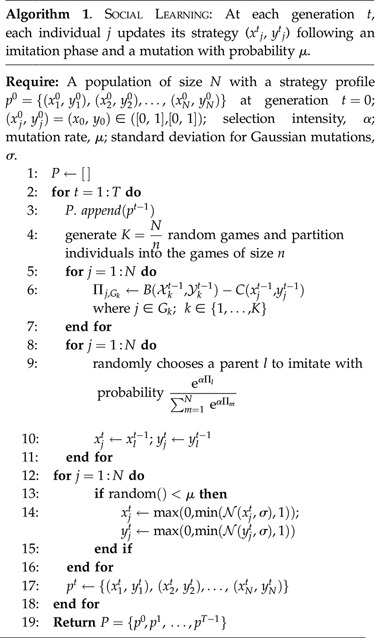


Finally, replicator dynamics and cross-learning dynamics are identical in the continuous-time limit [82]. Cross-learning is a particularly simple form of individual-based reinforcement learning that essentially coincides with the well-established threshold reinforcement model of social insects task allocation. Our model thus has a dual interpretation as a population of agents that individually learn by threshold reinforcement. A spectrum of other learning mechanisms has been identified to exhibit identical asymptotic properties [83] so that equivalences to other mechanistic models of learning could in principle be established. The details of this are, however, unfortunately beyond the scope of the current paper.

## 4. Results

We study the behaviour of the model discussed above in dependence on the parameters *b*_1_, *b*_2_, *w* and *β* that capture the characteristics of the environment (see table 1). Full details of the explored parameter sets can be found in electronic supplementary material, appendix S2.

**Table 1.** Model parameters

notation	definition	interpretation	values
*b*_1_	linear benefit coefficient for task *X* (e.g. thermoregulation)	efficiency of regulation; large values indicate easy regulation	(12, 30)
*b*_2_	quadratic benefit coefficient for task *X*	shape of benefit for task *X* (homeostatic versus maximizing)	( − 10, 10)
*β*	slope of benefit for task *Y* (e.g. brood care)	larger values indicate higher efficiency per unit of work	1, 2, …, 5
*w*	inflection point of benefit for task *Y*	1/*w* indicates minimum amount of work required	(0.1, 0.5)
*n*	group size for individual interactions		5
*α*	selection intensity in social learning		2
*μ*	mutation rate	probability of behaviour exploration	0.01
*σ*	mutation size	amount of behaviour variation	0.005
*T*	simulation end time		30 000

We are particularly interested in the conditions that determine whether a part of the population becomes inactive, effectively acting as free-riders. Formally, this corresponds to a branching of the population into two different groups of values on the trait *z*. In this situation the workload is not evenly shared across the population. Instead the sub-population with the lower *z* value benefits from the higher investment of the rest of the population. In the extreme case, one part of the population can be completely inactive (*z* = 0).

Our analysis starts from the complete model *M* that, as described above, consists of two prototypical tasks (foraging and brood care), combined with the option to remain passive. Subsequently, we decompose this model into two simpler sub-systems *M*_1_, *M*_2_, each capturing only one of the tasks combined with the possibility of remaining passive.

We use individual-based simulations to study the behaviour of the three systems for a broad range of environment types. Comparing the sub-systems *M*_1_ and *M*_2_ with the complete system *M*, which can be understood as the composition of the two sub-systems $M=M_{1}\cup M_{2}$, we find that the complexity of the task set can be a determinant of the emergence of laziness. In the same environment type, all workers remain active for the simpler task choices, while a sizeable inactive sub-population emerges when all tasks have to be handled simultaneously.

Finally, we investigate the stability of the task allocation, i.e. the reaction of the system to perturbations, to confirm that this corresponds to empirical findings.

Electronic supplementary material, appendix S3 shows that our simulation results agree with a theoretical analysis based on adaptive dynamics, an approach that is widely used to analyse the dynamics of EGT models under the assumptions of infinite populations and small mutations.

### 4.1. Behavioural regions in a complex environment and emergence of laziness

**Figure 4.**
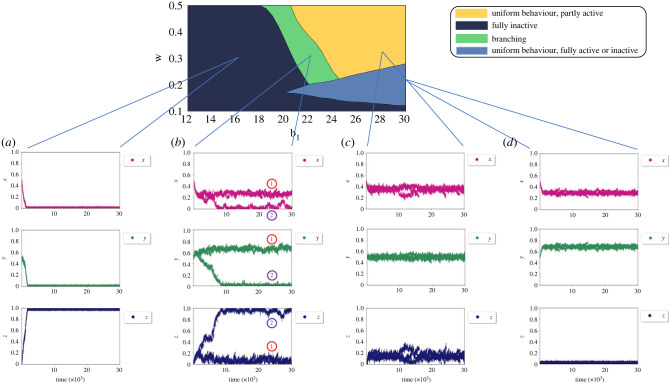
Simulation results of the system in different behavioural regions when *β* = 3 and *b*_2_ = −6. (*a*) *Fully Inactive* region: *b*_1_ = 16, *w* = 0.3. (*b*) *Branching* region: *b*_1_ = 23, *w* = 0.25. Each individual at every time step belongs to either one of the branches manually labelled red (1) or blue (2) in all the scatterplots. This is because only the combination of these branches allows *x* + *y* + *z* = 1. Here group (2) spend all of their time being inactive and are not engaged in doing tasks *X* or *Y*. The others (group (1)) divide their time between *X* (approx. 0.3) and *Y* (approx. 0.7) tasks with negligible inactive time. (*c*) *Uniform behaviour, partly active* region: *b*_1_ = 28, *w* = 0.3, where all individuals divide approximately all of their time between tasks *X* and *Y* with little time spent during inactivity. (*d*) *Uniform behaviour; fully active or inactive* region: *b*_1_ = 30, *w* = 0.2, where depending on the initial population trait values at *t* = 0, the population may develop towards full activity or full inactivity. For details of parameter definition and interpretations, see table 1 and for exploration of parameter effects on environment characteristics see electronic supplementary material, appendix S1.

**Figure 5.**
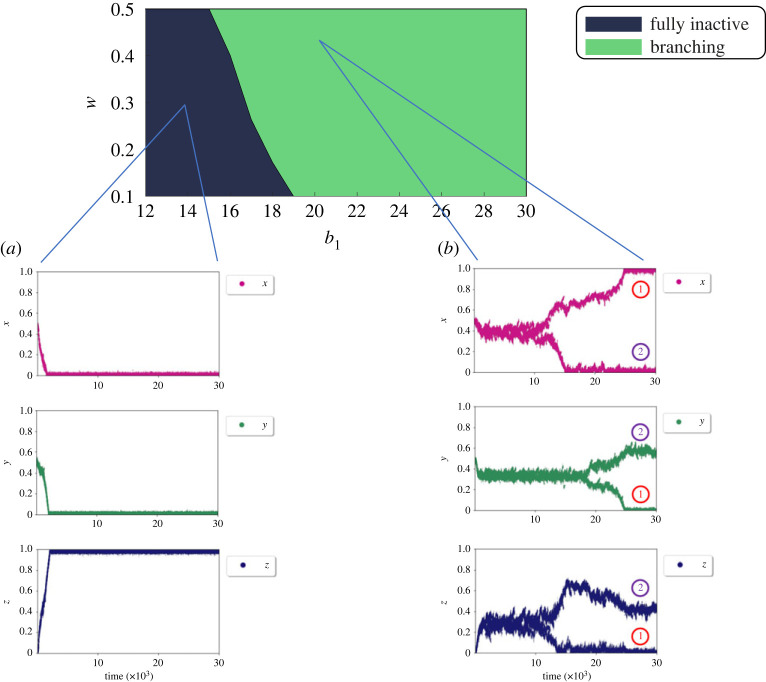
Simulation results of the system in different behavioural regions when *β* = 3 and *b*_2_ = −4. (*a*) *Fully Inactive* region: *b*_1_ = 14, *w* = 0.3; (*b*) *branching* region: *b*_1_ = 21, *w* = 0.45. Each individual at every time step belongs to either one of the branches manually labelled red (1) or blue (2) in all the scatterplots. This is because only the combination of these branches allows *x* + *y* + *z* = 1. Here, group (1) are fully active and specialized to task *X*. The other group (group (2)) are inactive approximately 0.4 of the time and doing *Y* for the rest with no engagement in task *X*. The branches in each trait scatterplot have been labelled with 1 and 2 to link the same sub-population branches between the three trait values given that for any individual we have *x* + *y* + *z* = 1. For details of parameter definition and interpretations, see table 1 and for exploration of parameter effects on environment characteristics see electronic supplementary material, appendix S1.

Figures 4 and 5 show the simulation results for the full model *M* in dependence on the parameters that capture the environment characteristics (table 1).^2^

The resulting space of behaviour variations can be divided into four different regions:

#### 4.1.1. Uniform behaviour; fully active or inactive

In this case, all individuals converge on a shared trait value, i.e. move towards a common level of engagement. The population may also develop towards full activity *z* = 0 or full inactivity *z* = 1, and whether this happens depends on the boundary conditions at *t* = 0. A single, shared trait value also means that the distribution of activity across the two tasks is identical for all individuals. This could be interpreted as a ‘fair sharing’ of the workload. The light blue region shows this behaviour.

#### 4.1.2. Uniform behaviour, partly active

In this case, all individuals move toward a shared level of engagement (i.e. a shared trait value 0 < *z* < 1) and uniform investment across the two tasks. The yellow region exhibits this behaviour. From the EGT perspective, this represents an evolutionarily stable strategy (ESS).

#### 4.1.3. Branching

After initial movement toward a shared level of engagement, the population splits into two (or more) coexisting traits (i.e. evolutionary branching). These sub-populations show different levels of investment (different *z* trait values). The distribution of energy across the two tasks is also different between the two groups. This could be interpreted as a part of the population ‘free-riding’ at the expense of the other individuals. The green region represents this kind of behaviour. Electronic supplementary material, appendix S3 discusses the mathematical conditions that need to be satisfied for an evolutionary branching strategy to arise.

#### 4.1.4. Fully inactivity

The entire population uniformly develops towards full inactivity (*z* = 1) and becomes inviable regardless of the boundary conditions. The dark blue region represents this behaviour.

Figures 4 and 5 show these findings. Note that regions and their boundaries are derived analytically from the adaptive dynamics in electronic supplementary material, appendix S3 and coloured accordingly. The individual trajectories shown in the panes below the region plot illustrate representative examples and typical behaviour. A more detailed exploration is given in electronic supplementary material, appendix S5.

It is evident that the environmental parameters determine the population steady state. As the regulatory task becomes more demanding (*b*_2_ = −4, figure 5), the regions of uniform behaviour disappear and the other regions are reshaped. In each of these settings, *b*_1_ and *w* determine the qualitative behaviour of the population.

An important question is how efficient any given behaviour is in terms of overall colony performance, captured by the total benefit obtained. Analysing this question allows us to tie the discussion back to the ultimate reasons of the behaviour. In particular, it allows us to investigate the question whether any given behaviour in our simplified model world gives rise to a fitness advantage.

*Relative colony efficiency* represents the ratio between the average payoff achieved and the optimal workforce allocation which maximizes the payoff. A ratio of 1 indicates optimal regulation. This concept is equivalent to the *price of anarchy* widely used in analysing the costs of decentralized organization [84].

**Figure 6.**
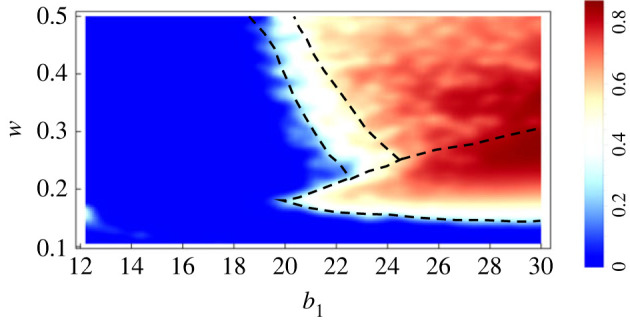
Relative colony efficiency. The ratio between the mean of the individual payoffs in the population’s selected strategy and the optimal theoretically achieved level in any given environmental condition. The relative colony efficiency can be between 0 and 1. The closer this measure is to 1, the closer the population strategy is to the optimal status. Parameters are selected similar to figure 4.

To determine the optimal workforce allocation, we use a stochastic global optimization meta-heuristic, differential evolution [85].^3^ Figure 6 shows the relative colony efficiency for the relevant parameter range given the same initial conditions as in figures 4 and 5 for the population traits. Note that the region labelled ‘uniform behaviour; fully active or inactive’ contains samples with high efficiency as well as samples with low efficiency. In general, the final fate of each individual point in this region depends on the initial conditions. However, we know analytically that this region will always contain a mix of fully active and fully inactive populations (see electronic supplementary material, appendix S3). It is evident that the branching region is not optimally regulated. In other words, the ‘price of anarchy’ has to be paid when a sub-population does not invest their full energy at the expense of others.

As an additional evaluation of the colony’s performance efficiency, we also investigated the social efficiency deficit (SED) measure which can generalize the concept of dilemma strength to social dilemmas of almost any complexity. SED is defined as the difference between an individual’s average payoff attained at the social optimal workforce allocation and at equilibrium emerging through evolutionary dynamics [86,87]. The results of this efficiency analysis are included in electronic supplementary material, appendix S4 and validate the extracted regions in figure 6 being qualitatively similar.

### 4.2. Behaviour in the simplified sub-systems

**Figure 7.**
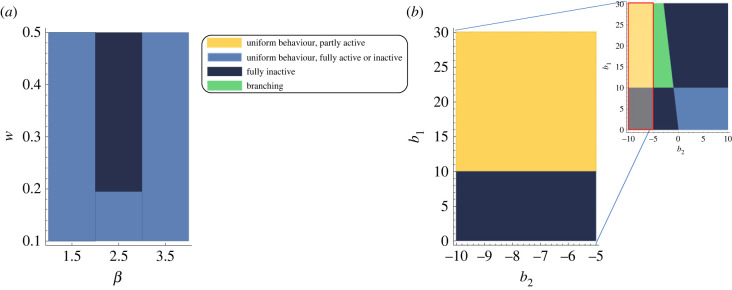
Environment regions for two sub-systems. Parameter exploration of these two sub-systems validates the choice of parameters in the regions of interest for the original system. (*a*) Environment regions for *M*_1_ and varying *w* and *β* illustrating different evolutionary behaviours when social learning rules are applied. *β* appears to have far less significant effects on the final fate of the workforce compared with *w*. Note that *β* = 1 is where in fact the benefit function is linear rather than being threshold-like and hence is not of interest in this work. (*b*) Environment regions for *M*_2_ and varying *b*_1_ and *b*_2_ illustrating different evolutionary behaviours when social learning rules are applied. Different regions may represent tasks with different natures such as a homeostatic task (which is of our interest here) or a maximizing task.

We turn to analysing the behaviour that emerges in the two simpler sub-systems *M*_1_ and *M*_2_. Figure 7 shows the behavioural regions of these two sub-systems for the relevant parameter range.

Each system *M*_*i*_ captures the choice between the *i*th task and (partial) inactivity in isolation.

For *M*_1_, figure 7*a*, explores the full space of the parameters *w* and *β* that determine the shape of the thresholding benefit function. No branching occurs in this sub-system for any of the parameter combinations.

For *M*_2_, figure 7*b* explores the parameter space of *b*_1_ and *b*_2_ within which the benefit function can represent a homeostatic task with unimodal benefit function or a task with monotonically increasing benefit, such as a maximizing foraging task (cf. electronic supplementary material, figure S1.a).

While all qualitative behaviour types are present in *M*_2_, no branching occurs for *b*_2_ ≤ −5 regardless of the value of *b*_1_.

Yet, in the full system *M* branching occurs for cost and benefit parameters in which neither of the sub-systems shows branching (figure 4). The reason for this is that the introduction of the choice between two tasks with different characteristics introduces a dilemma, namely to choose between (a) producing very high benefits from the brood-care task (high *y*) and (b) bearing minimum costs from engaging in this task (low *y*). Social dilemmas can spontaneously diversify a well-mixed population into stably coexisting groups of contributors [88] depending on the dilemma strength when exploring the parameter space [89]. Traditional models of social dilemma such as the Prisoner’s Dilemma and the snowdrift game [90] focus on the case of restricting the population to two distinct strategies while more recent works extend this to embody a continuous range of investments into a public good [91] as we do here.

**Figure 8.**
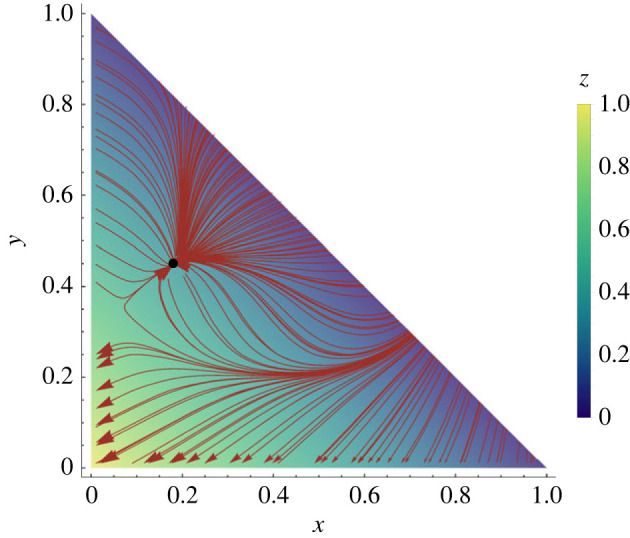
Streamline plot for *M* with *β* = 3, *w* = 0.3, *b*_1_ = 20 and *b*_2_ = −6. This setting is equivalent to the branching region in figure 4. The diagram depicts the evolution of a monomorphic population to the branching point, which agrees with the branching point found in the simulations.

Figure 8 shows the analytically derived population development to the branching point based on adaptive dynamics (see electronic supplementary material, appendix S1). This confirms that our simulation results are in agreement with the analytic treatment.

### 4.3. Colony reaction to worker loss: autonomous population change

**Figure 9.**
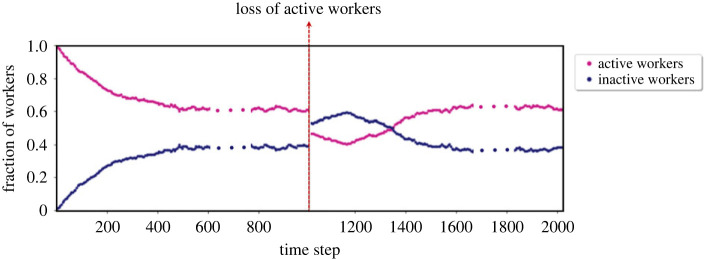
The ratio of the active/inactive population with respect to the whole population before and after the 20% drop in the number of active individuals.

An interesting question, resulting from the hypothesis that inactive workers form a ‘reserve workforce’ for emergency situations, is how the colony reacts to changes of the composition of the workforce. This has been investigated experimentally with culling experiments in which a part of the active or inactive workforce, respectively, is removed [65]. We can emulate such experiments virtually by running our model to steady state and removing a percentage of the active agents after the steady state has been reached. Figure 9 shows the development of worker proportions for such an experiment. After the removal of part of the active workforce at *t* = 1000 the proportions of active and inactive workers are briefly reversed, however, the colony quickly restores the same steady state. This is consistent with the experimental findings. Note that this is also analytically supported by the streamline plot in figure 8—the locally stable attractor guarantees that the population will return to the steady state as long as the perturbation is within limits.

## 5. Discussion

We have presented a new modelling approach for task allocation in social insect colonies to explain the presence of inactive workers. Our model is based on learning game theory (or EGT) and captures the dependence of behavioural changes on environmental factors. Most previous literature in the field does not consider this aspect in a principled form.

Our results suggest that the environmental conditions, modelled via cost and benefit functions, drive the behavioural patterns. Specifically, they can determine whether an inactive sub-population emerges. The model furthermore suggests that the complexity of task choices may play an important role. While workers do not become inactive in a simple task choice scenario, the same environmental factors cause an inactive sub-population to emerge when the choices become more complex.

This may provide important guidance for empirical investigations as it indicates that reducing the complexity of choices in experimental assays for task allocation may lead to *qualitatively* different outcomes.

On the theoretical side, we have only scratched the surface of what the EGT-based framework can afford us.

Our model differs from standard public goods games by the use of continuous traits. This enables us to capture partial inactivity not only on the colony level but also on the individual level. The possibility of reduced individual activity changes not only the equilibrium properties but also the behaviour dynamics. A standard nonlinear public good game would either have an internal equilibrium with a certain ratio of cooperator and defector individuals or show full cooperation/full defection over the entire population. In contrast, the proposed model with continuous-valued traits allows for various behavioural regions that show a richer spectrum of possibilities rather than just the extreme ends of behavioural regions (fully active or fully inactive). Our model also obtains regions where a stable coexistence of activity and inactivity (reduction of individuals’ general activity level, independent of task choice) may arise. Biologically relevant examples of public goods games without assortment that display such coexistence are very rare, and our model suggests that social insect colonies may be one of them.

As is to be expected, the shape of the benefit functions can have a significant influence on the outcomes. In the present paper, we have only analysed the behaviour of two particular types of benefit functions as a starting point: a thresholding task and a homeostatic task. We have performed preliminary analyses with other function shapes, specifically the combination of maximizing tasks, such as foraging, and homeostatic ones. The results indicate that thresholding benefits are more likely to generate the inactivity observed here. However, further systematic analysis of this is still required. An extensive catalogue of behaviour dynamics by tasks and benefit types is beyond the scope of the current paper. Here, we simply aim to introduce the general approach and to establish that environmental factors and task choice complexity can be determinants for the emergence of inactivity.

It is well established that qualitative classification of game dynamics by means of their cost and benefit functions is a strong tool in modelling long-term behaviour [92]. The exact quantitative nature of real biological cost and benefit functions can generally not be obtained easily. For this reason, a qualitative perspective such as this framework is a powerful mechanism to focus the discussion on different fundamental types of tasks. This perspective paves new paths to central questions in task allocation for both theoretical and experimental work.

An important insight suggested by our study, beyond the technical details, is that a plausible explanation for inactivity in social insect colonies can be given without having to invoke a ‘purpose’ of inactivity. It may simply be an epiphenomenon, an unavoidable by-product of the task allocation process. This possibility is neither implausible nor entirely surprising when one compares with the literature on human behaviour.

So-called ‘free-riding’ is widely observed in human society. The paradigmatic model of free-riding is the snowdrift game [56]. Free-riding is a close relative to the non-cooperation arising in the Prisoner’s Dilemma [93], the best known model to study the emergence of cooperation. The game-theoretic approach helps to distil the difference. In the Prisoner’s Dilemma, non-cooperation is always the best choice from the individual perspective. In the snowdrift game, however, the best course of action (to contribute or not to contribute) depends on the actions of the other participants. If nobody participates in the collective action, no shared benefit at all will be produced. The Prisoner’s Dilemma thus models the choice whether to actively disadvantage someone else for one’s own benefit, whereas the snowdrift game models the choice whether to fairly contribute to the cost of producing a shared benefit [90].

What is common to both scenarios and our study is that not contributing to the production of a shared benefit (non-cooperation) is not beneficial for the group outcome. Yet, it arises in the absence of a central coordination mechanism from individual-based decisions. It is important to appreciate that this individual-based decision making does not have to be the outcome of cognitively complex processes but can arise as a simple reaction to the limited proxy indicators available to the individual to assess its task performance.

The literature on eusociality and how social insects have managed to avoid ‘selfish’ behaviour detrimental to the colony’s good overall is vast and has been widely debated for decades (a comprehensive discussion from one perspective is provided in [94]). It is important to re-emphasize that this literature is focused on the evolutionary aspects (and reproduction), whereas our paper is not concerned with evolution and purely deals with the behavioural dynamics in a given evolutionary state. Generally, based on evolutionary considerations, free-riding should indeed not exist in social insect colonies at all or only to a very minor degree, unless, of course, it offers hidden fitness benefits. Empirically we do, however, know that inactivity (or ‘laziness’) exists in significant amounts. Our present work is concerned with the question: why is this the case if an evolutionary view says it should have been avoided? Do we have to assume other evolutionary pressures (such as the ‘reserve workforce’ hypothesis) that assign inactivity a fitness benefit? Our answer is that this is not necessarily the case. The key is that, generally speaking, it stands to reason that individuals must evaluate and take into account individual-level costs to some degree in their decisions. This is unavoidable because, without considering individual costs, neither an individual nor a whole colony would be able to optimize their choices (individual costs contribute to social cost). Furthermore, other behaviours where individuals occasionally preference individual benefits are well-known and have attracted interest for a long time, for example, reproductive conflict and egg-laying by workers [95]. Our behavioural model shows that, once this is taken into account, inactivity can emerge in some settings as a side effect, even where this can be detrimental to the overall colony benefit.

A hypothesis such as the one put forward here ultimately requires experimental verification. A promising approach is to test whether the behaviour dynamics can be changed as predicted by manipulating task costs and benefits, and we are currently undertaking such experiments.

## Data Availability

All codes and materials are publicly available from GitHub: https://github.com/Moein-Khajehnejad/Inactivity-in-Social-Colonies. The data are provided in electronic supplementary material [96].

## Endnotes

1 Note, however, that this is not universally true for all tasks and in all species [79].

2 The source code of the simulations is available at this GitHub link: https://github.com/Moein-Khajehnejad/Inactivity-in-Social-Colonies.

3 As implemented in Scipy 0.19.0.
